# Simvastatin Reduces Endotoxin-Induced Acute Lung Injury by Decreasing Neutrophil Recruitment and Radical Formation

**DOI:** 10.1371/journal.pone.0038917

**Published:** 2012-06-11

**Authors:** Jochen Grommes, Santosh Vijayan, Maik Drechsler, Helene Hartwig, Matthias Mörgelin, Rolf Dembinski, Michael Jacobs, Thomas Andreas Koeppel, Marcel Binnebösel, Christian Weber, Oliver Soehnlein

**Affiliations:** 1 Institute for Molecular Cardiovascular Research (IMCAR), RWTH Aachen, Aachen, Germany; 2 European Vascular Center Aachen-Maastricht, RWTH Aachen, Aachen Germany and Maastricht University Medical Center, Maastricht, The Netherlands; 3 Institute for Cardiovascular Prevention, Ludwig-Maximilians-University Munich, Munich, Germany; 4 Division of Infection Medicine, Department of Clinical Sciences, Lund University, Lund, Sweden; 5 Department of Intensive Care Medicine, RWTH Aachen, Aachen, Germany; 6 Department of General, Visceral and Transplantation Surgery, RWTH Aachen, Aachen, Germany; 7 Cardiovascular Research Institute Maastricht, Maastricht University, Maastricht, The Netherlands; Louisiana State University, United States of America

## Abstract

**Introduction:**

Treatment of acute lung injury (ALI) remains an unsolved problem in intensive care medicine. As simvastatin exerts protective effects in inflammatory diseases we explored its effects on development of ALI and due to the importance of neutrophils in ALI also on neutrophil effector functions.

**Methods:**

C57Bl/6 mice were exposed to aerosolized LPS (500 µg/ml) for 30 min. The count of alveolar, interstitial, and intravasal neutrophils were assessed 4 h later by flow cytometry. Lung permeability changes were assessed by FITC-dextran clearance and albumin content in the BAL fluid. *In vitro*, we analyzed the effect of simvastatin on neutrophil adhesion, degranulation, apoptosis, and formation of reactive oxygen species. To monitor effects of simvastatin on bacterial clearance we performed phagocytosis and bacterial killing studies *in vitro* as well as sepsis experiments in mice.

**Results:**

Simvastatin treatment before and after onset of ALI reduces neutrophil influx into the lung as well as lung permeability indicating the protective role of simvastatin in ALI. Moreover, simvastatin reduces the formation of ROS species and adhesion of neutrophils without affecting apoptosis, bacterial phagocytosis and bacterial clearance.

**Conclusion:**

Simvastatin reduces recruitment and activation of neutrophils hereby protecting from LPS-induced ALI. Our results imply a potential role for statins in the management of ALI.

## Introduction

Acute lung injury (ALI) is a life-threatening disease with an age-adjusted incidence of 86.2 per 100.000 person-years [1]. Despite all innovations in intensive care medicine, the mortality of ALI remains up to 40%. ALI is characterized by an increased permeability of the alveolar-capillary barrier resulting in lung edema with protein-rich fluid consequently leading to impairment of arterial oxygenation. A major cause for development of ALI is sepsis, wherein Gram-negative bacteria are a prominent cause [2]. LPS inhalation mimics human Gram-negative ALI, inducing neutrophil recruitment, pulmonary edema and finally impairment of gas exchange [3].

Recruitment of neutrophils is a key event in development of ALI resulting in plasma leakage and deterioration of oxygenation [1], [4]. The importance of neutrophils in ALI is affirmed by studies where lung injury is abolished or reversed by depletion of neutrophils [5], [6]. Granule proteins from activated neutrophils, *e.g.* azurocidin and α-defensins, alter directly permeability changes [7], [8]. Moreover, proteases of neutrophilic origin such as neutrophil elastase have been regarded to be important in degradation of surfactant proteins, epithelial cell apoptosis, and coagulation [9], [10]. In addition, neutrophils produce vast quantities of reactive oxygen (ROS) and nitrogen (RNS) species like O_2_^• −^ and NO ^•^ through their oxidant-generating systems such as the phagocyte NADPH oxidase and nitric oxide synthase (NOS), respectively. Besides their important antimicrobial effector function, neutrophil-derived oxidants promote deleterious pro-inflammatory effects thus being a major cause of neutrophil-dependent tissue injury in ALI [4]. Although a large body of evidence indicates the importance of neutrophils in ALI, it should also be pointed out that ALI or acute respiratory distress syndrome (ARDS) can also occur in patients with neutropenia [11], [12].

Statins, inhibitors of 3-hydroxy-3-methylglutaryl coenzyme A (HMG-CoA) reductase, are a class of drugs used for their lipid-lowering effects and thereby preventing cardiovascular disease. However, recent studies have revealed anti-inflammatory pleiotropic effects and plaque stabilizing effects of statins [13]. Their beneficial effects cannot entirely attribute to reduction of lipid levels [14], [15]. Studies on the effect of statins in ALI have repeatedly shown attenuation of vascular leakage various models of ALI [16], [17], an effect predominantly attributed to endothelium-protecting effects of statins. However, with the importance of neutrophils in ALI and in regulation of vascular permeability [18], we here investigate effects of statins on neutrophil function.

Although previous *in vitro* and *in vivo* studies have revealed the beneficial effects of statins in ALI, little is known about the effects of statins on neutrophils in ALI. Beside the beneficial effect of lovastatin in acute lung injury, *Fessler* et al. revealed impaired host defence due to inhibitory effects on Rac activation, actin polymerization, chemotaxis, and bacterial killing of neutrophils isolated from lovastatin-treated mice [19]. Recruitment of neutrophils, release of granule proteins and generation of ROS by neutrophils display key events in ALI and may represent a potential target for therapy. Consequently, we address the effect of simvastatin treatment on ALI and on neutrophil effector functions.

## Methods

### Animals

Male C57Bl/6 mice, 8 weeks of age, were obtained from Janvier (Elevage Janvier, Le Genest St. Isle, France). Neutrophils were depleted by intraperitoneal injection of monoclonal antibody 1A8 (100 µg per mouse 12 hours and 0 hours before *Lipopolysaccharid* (LPS) inhalation, BioXcell, West Lebanon, N. Hamp.). Mice were treated with simvastatin (2 µg/g bodyweight) or NaCl 0.9% by intraperitoneal injection 12 hours and 0 hours before LPS inhalation or 1 hour after LPS inhalation, respectively. The experiments were officially approved by the state office for nature, environment and consumer protection Cologne, Germany (Landesamt für Natur, Umwelt und Verbraucherschutz Köln, AZ 87-51.04.2010.A268). All animals received humane care in accordance with the requirements of the German Animal Protection Act (Tierschutzgesetz), §8 Article 1 and in accordance to the Guide for the Care and Use of Laboratory Animals published by the German National Institute of Health.

### Murine Model of Acute Lung Injury

Murine model of ALI was performed as described previously [20], [21]. Aerosolized LPS from *Salmonella enteritidis* (Sigma Co., St. Louis, MO) dissolved in 0.9% saline (500 µg/ml) was utilized to induce neutrophil-infiltration in the lung. Six mice were exposed simultaneously to aerosolized LPS in a custom-built box (22 cm in length; 10 cm in diameter) connected to an air nebulizer (MicroAir, Omron Healthcare, Vernon Hills, IL) for 30 minutes. Control mice were exposed to saline aerosol. Neutrophil counts in bronchoalveolar lavage (BAL) and lung tissue (interstitium and pulmonary vasculature) were assessed 4 hours after inhalation. 30 min before euthanasia, 5 µl FITC-Ly-6G anti-mouse (eBioscience) labeling intravascular neutrophils and 100 µl Fluorescein isothiocyanate-Dextran (30 mg/ml FITC-Dextran; 70 kDa, Sigma-Aldrich) were applied by tail vein injection. The mice were anesthetized with an intraperitoneal injection of ketamine (125 mg/kg body weight; Sanofi-Cefa GmbH Düsseldorf, Germany) and xylazine (12.5 mg/kg b.w.; Phoenix Scientific). The trachea was dissected and cannulated (PortexFineBore Polythene Tubing, 0.28 mm inner diameter (ID)/0.61 mm outer diameter (OD), Smiths Medical International, Keene, NH). 5×0.5 ml PBS was injected and withdrawn. Thereafter, the ribcage was opened by a midline incision and the pulmonary vasculature was rinsed with 15 ml ice-cold PBS with 0.5 mM EDTA after cutting the inferior cava vein to facilitate exsanguination. The lungs were removed, minced and digested with Liberase (1:20; 25 mg Liberase RI/ml aqua, Roche Mannheim Germany). Digested lungs were passed through a cell strainer (70 µm; MiltenyiBiotec GmbH, Bergisch Gladbach, Germany) and the resulting single cell suspension was centrifuged for 5 min at 300 g. The pellets were resuspended in 1 ml hank’s balanced salt solution with 0.3 mmol/l EDTA and 0.1% BSA. The bronchoalveolar lavage (BAL) fluid was centrifuged for 5 min at 300 g [20], [21].

### Flow Cytometry

Cell pellets were labeled with PerCP-Cy5.5 anti-mouse Ly-6G, PE anti-mouse CD115, APC-Cy7 anti-mouse CD45 und APC anti-Mouse F4/80 (all eBioscience). Neutrophils were identified by their typical appearance in the forward scatter-side scatter and as CD45^+^ CD115^–^ and PerCP- Gr1^+^ cells [20], [21]. Within the lung, FITC-Gr1 antibody was used to distinguish between interstitial neutrophils (CD45^+^, CD115^–^, PerCP- Gr1^+^, FITC-Ly6G ^-^) and intravasal neutrophils (CD45^+^, CD115^–^, PerCP- Gr1^+^, FITC-Ly6G ^+^) as described previously [20], [22]. All studies were performed on a FACS Canto II (Becton Dickinson, San Jose, CA) and data were analyzed using FlowJo software (Tree Star, Ashland, OR).

### Lung Permeability

FITC-Dextran (70 kDa, Sigma-Aldrich) was used to assess vascular leakage. 100 µl FITC-Dextran (30 mg/ml) were administered by tail vein injection 30 min prior to euthanasia and dye extravasation was used to assess change in vascular permeability. The fluorescence of the 100 µl BAL supernatant (Fluo_BAL_) and of 50 µl serum (Fluo_Serum_) was measured and permeability volume was expressed in microlitre (V_Perm_ = (Fluo_BAL_/100 µl)/(Fluo_Serum_/50 µl) * BAL volume) [20], [21].

### Albumin Concentration of the BAL

The albumin content of the BAL supernatants was assessed using ELISA Kit for Albumin (E91028Mu Uscn Life Science Wuhan China). Measurement of absorbance at 450/540 nm was performed with a microplate reader (infinite 200,Tecan Group Switzerland).

### Elastase Acitivity

Elastase activity was measured using the Molecular Probes’ EnzChek® Elastase Assay Kit (E-12056 Molecular Probes Europe, Leiden, The Netherlands). Measurement of absorbance at 515 nm was performed with a microplate reader (infinite 200,Tecan Group Switzerland).

### Histology and Electron Microscopy

After completion of the experiment, one part of the right lung was fixed in formalin, embedded in paraffin wax and stained with Mayer’s haematoxylin and eosin for histological examination. Another part of the lung was prepared for scanning electron microscopy as described [6]. We scored histological sections based on a scheme outlined in a recent report from the American Thoracic Society outlining standardized guidelines for models of ALI in animals [23].

### Neutrophil Isolation

Human neutrophils were isolated and purified from venous blood of healthy donors by density gradient centrifugation using Polymorphprep™ system (Axia-Shield, Fisher-Scientific, Schwerte Germany). 20.0 ml of EDTA-anticoagulated whole blood was layered over 20 ml of Polymorphprep™ in a 50 ml centrifuge tube. Centrifugation (450–500×g for 30–35 minutes in a swing-out rotor at 18–22°C) resulted in separation with two leucocyte bands visible, whereof the lower one represented the neutrophils. Polymorphonuclear cells were washed and resuspended in culture medium (RPMI Medium invitrogen) [24].

### Degranulation

After incubation with Simvastatin, neutrophils were activated by adding 10 µM fMLP (N-Formylmethionyl-Lencyl-Phenylalanin) (Sigma) and upregulation of CD11b, CD29 and FPRL1 was measured after 30 min using FACS Canto II.

### Flow Chamber

We coated petri dishes with fibronectin or ICAM1 (1 µg/ml +10% BSA) for laminar flow chamber. Neutrophils were treated with Simvastatin (1 µM or 10 µM for 3 hour respectively). After activation with 10 µM fMLP (Sigma), neutrophils were perfused at 1 dyne/cm^2^ over fibronectin or ICAM1 and firmly adherent neutrophils were quantified after 4 min in multiple fields (at least 6 fields, 100 × magnification).

### Reactive Oxygen Species

ROS was detected by dihydrodichlorofluoresceindiacetate (DCF, Molecular Probes, Eugene, OR USA) as described previously [25]. Basically, cells were incubated with the profluorescent, lipophilic H2-DCF-DA which can diffuse through the cell membrane. Reaction with intracellular ROS results in the fluorescent molecule DCF (max. emission ∼530 nm), so that DCF fluorescence can be used as a measure for intracellular ROS levels. Fluorescence intensity was quantified with FACS Canto every 10 min over 1 hour.

### Bacterial Killing

After incubation of neutrophils with simvastatin, neutrophils were incubated with *E. coli* (BL21). After 20 min, neutrophils were disrupted by alkaline lysis (H_2_O with NaOH at pH 14). Viable bacteria were then grown on Luria Bertani agar over night and the colonies enumerated.

### Phagocytosis

Fluorescent *E. coli* and opsonizing reagent (Molecular Probes) were reconstituted as indicated by the manufacturer. IgG opsonization was achieved according to the manufacturer’s instructions. Complement opsonization was attained by incubation of bacteria with fresh human serum at 37°C for 1 hour. Opsonized particles were washed and seeded onto neutrophils which had been incubated with Simvastatin (1 µM or 10 µM) for 3 hours. Neutrophils were activated by adding 10 µM fMLP. Fluorescence was measured with FACS Canto (Becton Dickinson, San Jose, CA) after 60 min.

### Apoptosis

After incubation with simvastatin, apoptosis of isolated neutrophils were was measured using Annexin V Kit (Annexin V Apoptosis Detection Kit I Cat.nr. 556570 BD). Neutrophils were resuspended in Annexin V binding buffer and stained with FITC-conjugated Annexin V antibody (BD Pharmingen Cat. Nr. 556419). After 15 minutes PerCP Cy7 conjugated 7-Aminoactinomycin (7AAD) (BD Pharmingen Cat.Nr. 559925) was added to distinguish between apoptotic and necrotic cells. Apoptosis was assessed after 3 hours and 24 hours by FACS analysis.

### *In vivo* Analysis of Degranulation after LPS Inhalation

LPS inhalation and sample preparation (BAL and lung) was done as described above. Degranulation was measured by *Flow cytometry*. Cell pellets were labeled with PerCP-Cy5.5 anti-mouse Ly-6G, PE anti-mouse CD115, APC-Cy7 anti-mouse CD45 und APC anti-Mouse Ly- 6G (all eBioscience) and FITC anti-mouse CD29 (Miltenyi Biotec). Neutrophils were identified by their typical appearance in the forward scatter-side scatter and as CD45^+^ CD115^–^ and PerCP- Ly-6G ^+^ cells. Within the lung, APC Ly-6G antibody was used to distinguish between interstitial neutrophils (CD45^+^, CD115^–^, PerCP- Gr1^+^, APC-Ly6G ^-^) and intravascular neutrophils (CD45^+^, CD115^–^, PerCP- Gr1^+^, APC -Ly6G ^+^).

In addition blood was withdrawn by retro orbital puncture 4 h after LPS inhalation respectively. 100 µl EDTA-blood was lysed for 15 min using lysing buffer. Cell pellets were labeled with PerCP-Cy5.5 anti-mouse Ly-6G, PE anti-mouse CD115, APC-Cy7 anti-mouse CD45 und APC anti-Mouse CD11b (all eBioscience) and FITC anti-mouse CD29 (Miltenyi Biotec). Degranulation of neutrophils were measured using FACS Canto (Becton Dickinson, San Jose, CA).

### *In vivo* Analysis of Bacterial Clearance

Cecal ligation and puncture (CLP) was conducted as described previously [3], [20]. After 24 hours, the mice were sacrificed with an intraperitoneal injection of ketamine (500 mg/kg body weight; Sanofi-Cefa GmbH Düsseldorf, Germany) and xylazine (50 mg/kg b.w.; Phoenix Scientific). The lungs were removed, harvested. The supernatant of the lungs (10 µl and 50 µl) were spread on Luria Bertani agar over night and the colony forming units (CFU) were enumerated. Mice were treated with simvastatin (2 µg/g bodyweight) or NaCl 0.9% by intraperitoneal injection 0 hours and 12 hours after CLP. In addition, we conducted a midline laparotomy without cecal ligation and puncture in the control group (sham operation).

### Statistics

All data are expressed as mean ± standard deviation. Statistical calculations were performed using GraphPad Prism 5 (GraphPad Software Inc.). ANOVA, unpaired Student’s t-test, or Kruskal-Wallis test with posthoc Dunn tests, Bonferroni post tests or Newman-Keuls Multiple Comparison test were used as appropriate. * indicates a *p*-value <0.05.

## Results

### Protective Role of Simvastatin Treatment Pre and Post Inducing ALI

**Figure 1:**
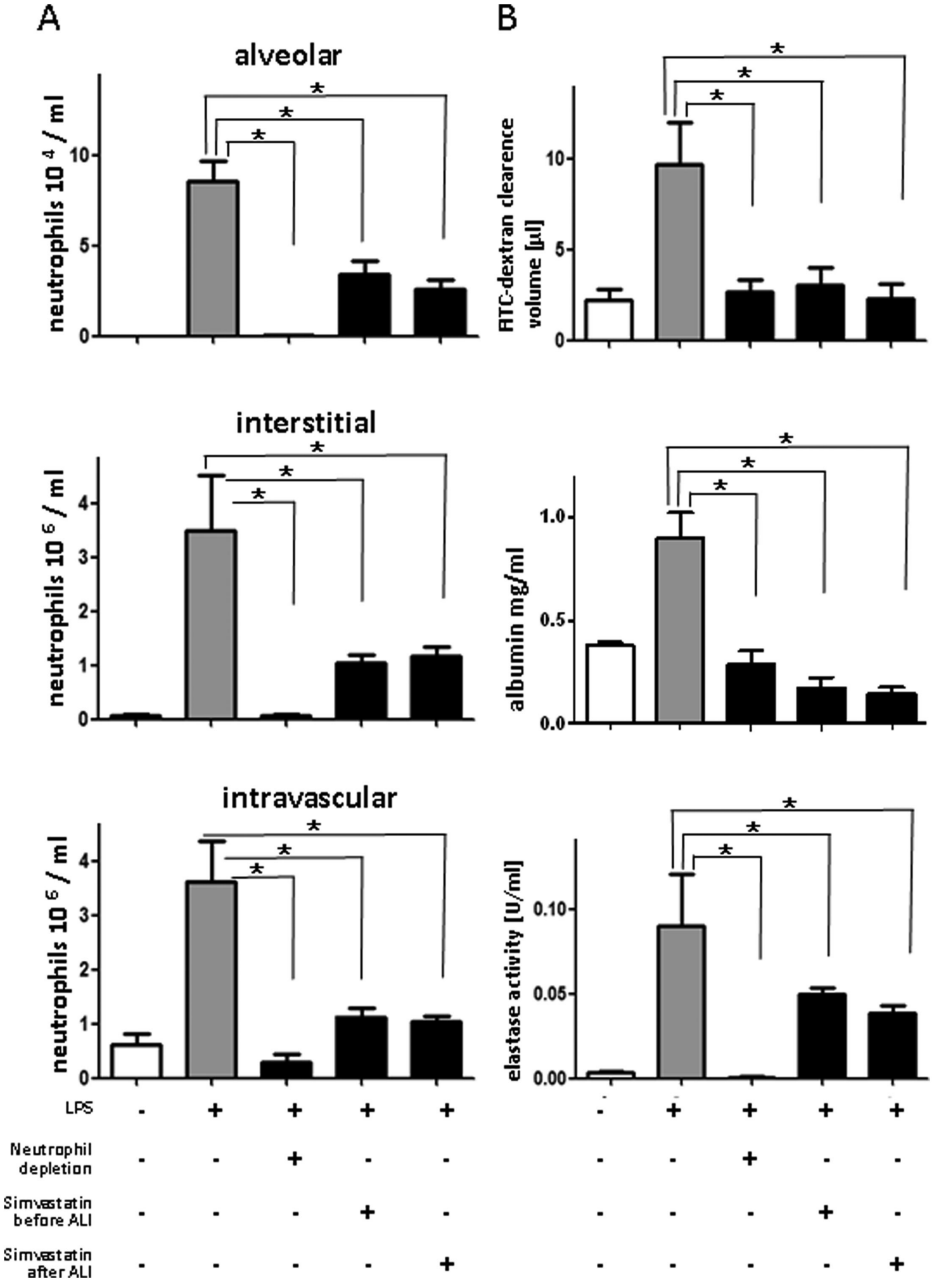
Simvastatin reduces LPS-induced acute lung injury by interference with neutrophil recruitment. Mice were challenged with LPS via inhalation and sacrificed 4 hours later. In addition, neutrophils were depleted by antibody injection or mice were treated with simvastatin (2 µg/g bodyweight) 12 hours and one hour before or one hour after LPS exposure as indicated. **A:** Quantification of alveolar (top), interstitial (middle), and intravascular neutrophils (bottom) in mice treated as indicated. **B:** FITC-dextran clearance (top), albumin concentration (middle), and elastase activity (bottom) in BAL fluids in mice treated as indicated (n = 8–10 for each bar). Statistical significance was tested using one way ANOVA with Newman-Keuls Multiple Comparison test. * indicates significant difference compared to LPS-treated animals.

LPS-inhalation increased the number of intravascular, interstitial, and alveolar neutrophils as analyzed by flow cytometry of lung homogenates and BALF (Fig. 1 A) [20], [21]. Furthermore, the albumin concentration as well as the clearance volume of the fluorescent dextran increased in the BALF by LPS treatment indicating enhanced plasma leakage and edema formation. Moreover, the activity of neutrophil-derived elastase was elevated in LPS-treated animals (Fig. 1B). The role of neutrophils in LPS-mediated ALI was assessed by selective depletion of neutrophils by antibody injection [26]. In line with the previously reported importance of neutrophils in ALI [4], we found that neutrophil depletion abolishes permeability increases, elastase accumulation (Fig. 1B), and structural changes induced by LPS (not shown).

Simvastatin treatment pre or post has the same impact in LPS-induced ALI as removing neutrophils. By treatment at either time point, neutrophil adhesion as well as interstitial and alveolar infiltration were largely abolished (Fig. 1A). Furthermore, simvastatin treatment reduced LPS-induced permeability changes to a degree similar to what was observed by neutrophil depletion (Fig. 1B) suggesting that simvastatin primarily acts via modulating neutrophil function. Finally, simvastatin treatment blocked accumulation of neutrophil elastase in the BALF (Fig. 1B).

**Figure 2:**
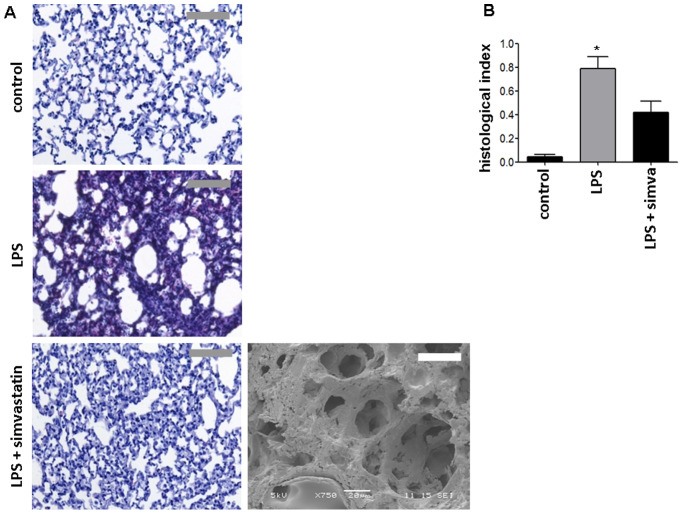
Simvastatin prevents LPS-induced structural changes in the lung tissue. Representative histological (left) and scanning electron microscopic (right) images of lungs from mice treated as indicated. Scale bars indicate 50 µm for scanning electron microscopy and 250 µm for histology. Quantification of histological lung sections (bottom) (n = 4 for each bar). Statistical significance was tested using ANOVA with Newman-Keuls Multiple Comparison test. * indicates significant difference compared to LPS-treated animals.

Histological and ultrastructural analyses of lungs following LPS exposure revealed alveolar septal thickening, accumulation of inflammatory cells in the interstitium and the alveoli, and influx of protein-rich fluid into the alveolar space as compared to control mice exposed to aerosolized NaCl 0.9% (Fig. 2). SEM images for control and LPS treatment were published previously [20], [21]. In addition, simvastatin both before (Fig. 2) and after LPS inhalation (data not shown) reduced structural changes provoked by LPS exposure.

### Simvastatin Reduce Adhesion of Neutrophils to ICAM-1 and Fibronectin

**Figure 3:**
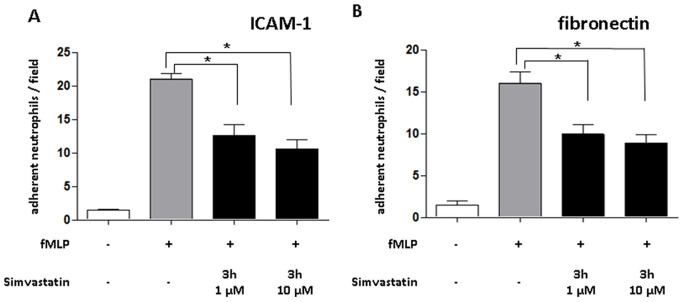
Simvastatin impairs neutrophil adhesion. Isolated human neutrophils were pre-treated with simvastatin (3 hours, 1 or 10 µM) and then activated with fMLP. Neutrophils were perfused over immobilized recombinant ICAM-1 or fibronectin at 1 dyne/cm^2^ and the number of adherent cells was enumerated. n = 8–10 for flow chamber experiments (repetition of the experiment = 4). Statistical significance was tested using one way ANOVA with Newman-Keuls Multiple Comparison test.* indicates significant difference of fMLP treated samples compared to each other samples.

To analyze the effect of simvastatin treatment on neutrophils, we treated isolated human neutrophils with simvastatin prior to perfusion over immobilized ICAM-1 (Fig. 3A) or fibronectin (Fig. 3B). Treatment of neutrophils with simvastatin for 3 hours (Fig. 3) at 1 or 10 µM severely diminished adhesion to either substrate indicating a direct effect on neutrophil adhesive functions.

### Simvastatin Reduces fMLP Induced ROS Formation of Neutrophils

**Figure 4:**
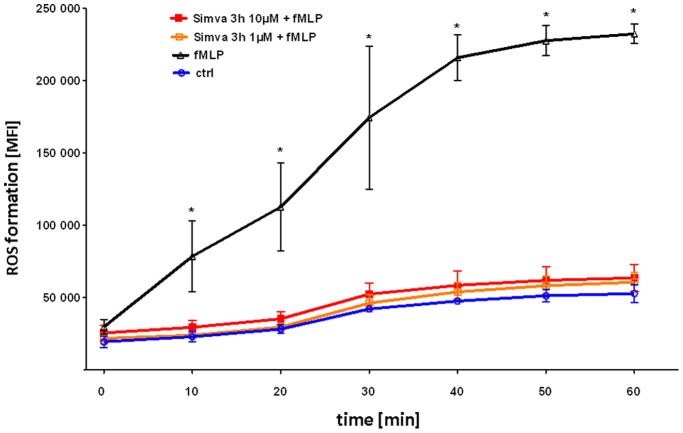
Simvastatin blocks ROS formation in neutrophils. Isolated human neutrophils were pre-treated with Simvastatin (3 hours, 1 or 10 µM), stained with ROS-sensitive H2-DCFDA and then activated with fMLP. In addition, neutrophils were pre-treated with simvastatin as indicated and fluorescence was measured by flow cytometry FACS Canto (Becton Dickinson, San Jose, CA) every ten minutes following fMLP stimulation. n = 6 for each group; repetition = 4. Statistical significance was tested using one way ANOVA with Newman-Keuls Multiple Comparison test. * indicates significant difference in comparison to the control group at each different time point.

We investigated the effect of simvastatin on ROS formation induced by fMLP, a secretagogue shed from bacterial walls. fMLP clearly induced formation of ROS over time (Fig. 4). Pretreatment of neutrophils with simvastatin for 3 hours abolished ROS formation.

### Effect of Simvastatin on Degranulation of Neutrophils

**Figure 5:**
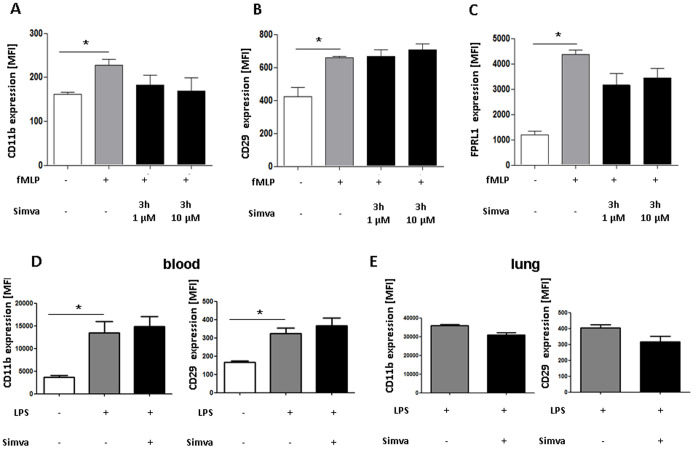
Influence of simvastatin on neutrophil degranulation *in vitro* (A–C) and *in vivo* (D+E). Isolated human neutrophils were pre-treated with Simvastatin (3 hours, 1 or 10 µM) and then activated with fMLP. MFI of surface expression of CD11b (A), CD29 (B) and FPRL1 (C) as measured by flow cytometry after staining with directly conjugated antibodies (n = 3–6; repetition = 4). **D+E** *In vivo* degranulation after LPS inhalation. Mice were challenged with LPS via inhalation and sacrificed 4 hours later. After isolation of neutrophils from the blood and the lung, MFI of surface expression of CD11b and CD29 as measured by flow cytometry after staining with directly conjugated antibodies (n = 4–6). Statistical significance was tested using one way ANOVA with Newman-Keuls Multiple Comparison test. * indicates significant difference in comparison to the fMLP or the LPS group.

Degranulation of neutrophils can be assessed by detection of secreted proteins or by FACS analysis of receptors incorporated into the cell membrane after granule discharge [27]. Here we went for the latter approach and investigated the upregulation of CD11b, CD29, and FPRL1 from intracellular stores following stimulation with fMLP (Fig. 5A–C). Treatment in this way significantly enhanced surface expression of all molecules. However, simvastatin failed to significantly reduce the effect of fMLP. Nevertheless, there was a trend to reduced expression of FPRL1 and CD11b. Similar data were also found in preliminary experiments where MPO and neutrophil elastase were assessed in the supernatant of activated and simvastatin treated neutrophils (data not shown).

To assess neutrophil-degranulation *in vivo*, we assessed neutrophil CD11b and CD29 expression after LPS inhalation. LPS inhalation induced a significant increase of both receptors on circulating neutrophils (Fig. 5D). However, their expression was not affected in mice treated with simvastatin (Fig. 5D). In interstitial lung neutrophils, however, simvastatin treatment allowed for a trend towards reduced expression of CD11b and CD29 (Fig. 5E).

### Simvastatin does not Impair Neutrophil Bacterial Killing

**Figure 6:**
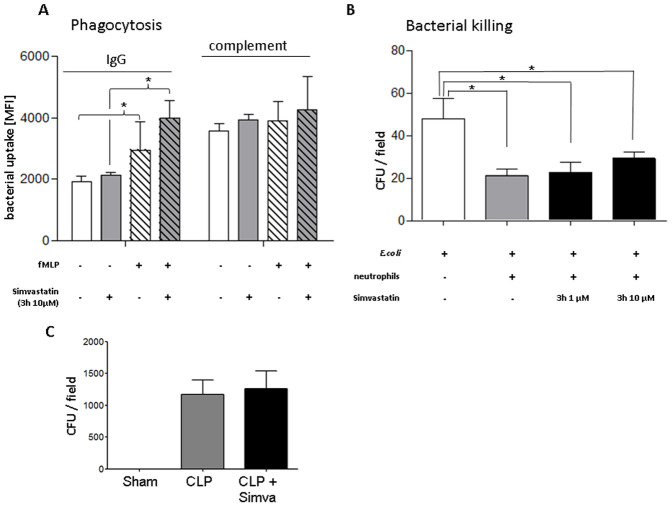
Simvastatin does not affect neutrophil antimicrobial activity *in vitro* (A + B) and *in vivo* (C). A: Bacterial uptake of fluorescent IgG- or complement-opsonized E. coli by activated or resting neutrophils as assessed by flow cytometry. Neutrophils were activated with fMLP and pretreated with simvastatin as indicated n = 4; repetition = 4. Statistical significance was tested using two way ANOVA with with Bonferroni posttest. * indicates significant difference to the control group without fMLP. **B:** Formation of colony forming units (CFU) after hypotonic lysis of neutrophils that had phagocytozed *E. coli* (BL21) for 20 min. (n = 4). **C** No negative effect on neutrophil bacterial clearance in acute lung injury. 24 h after cecal ligation and puncture (CLP), the supernatant of the harvested lungs was spread out on Luria Bertani agar over night and the colony forming units (CFU) were enumerated. The CFU did not increase after simvastatin treatment in comparison to the CLP group indicating no negative effect on the bacterial clearance (n = 5). Statistical significance was tested using one way ANOVA with Newman-Keuls Multiple Comparison test. * indicates significant difference to the control group without neutrophils.

To analyze negative anti-inflammatory effects of simvastatin we tested the capacity of simvastatin-treated neutrophils to phagocytose and clear bacteria. Compared to IgG-opsonization, complement-opsonization increased bacterial uptake (Fig. 6A). With igG opsonization bacterial uptake was increased following fMLP exposure. Simvastatin had no effect on the uptake of IgG- or complement-opsonized bacteria by activated or resting neutrophils (Fig. 6A). Finally, we investigated the ability of simvastatin to clear bacteria using a bacterial killing assay. To this end, neutrophils were allowed to phagocytose living *E. coli* (BL21) and clearance was assessed after alkaline lyses and subsequent enumeration of colony forming units (Fig. 6B).

To further test the impact of simvastatin treatment on bacterial clearance we induced sepsis by cecal ligation and puncture (CLP). After 24 hours lungs were collected, homogenized and colony forming units (CFU) following culture on LB agar over night were enumerated. As we did not observe increases in CFUs in mice receiving simvastatin (Fig. 6C), we conclude that simvastatin does not negatively impact on bacterial clearance.

### Simvastatin does not Affect Neutrophil Apoptosis

**Figure 7:**
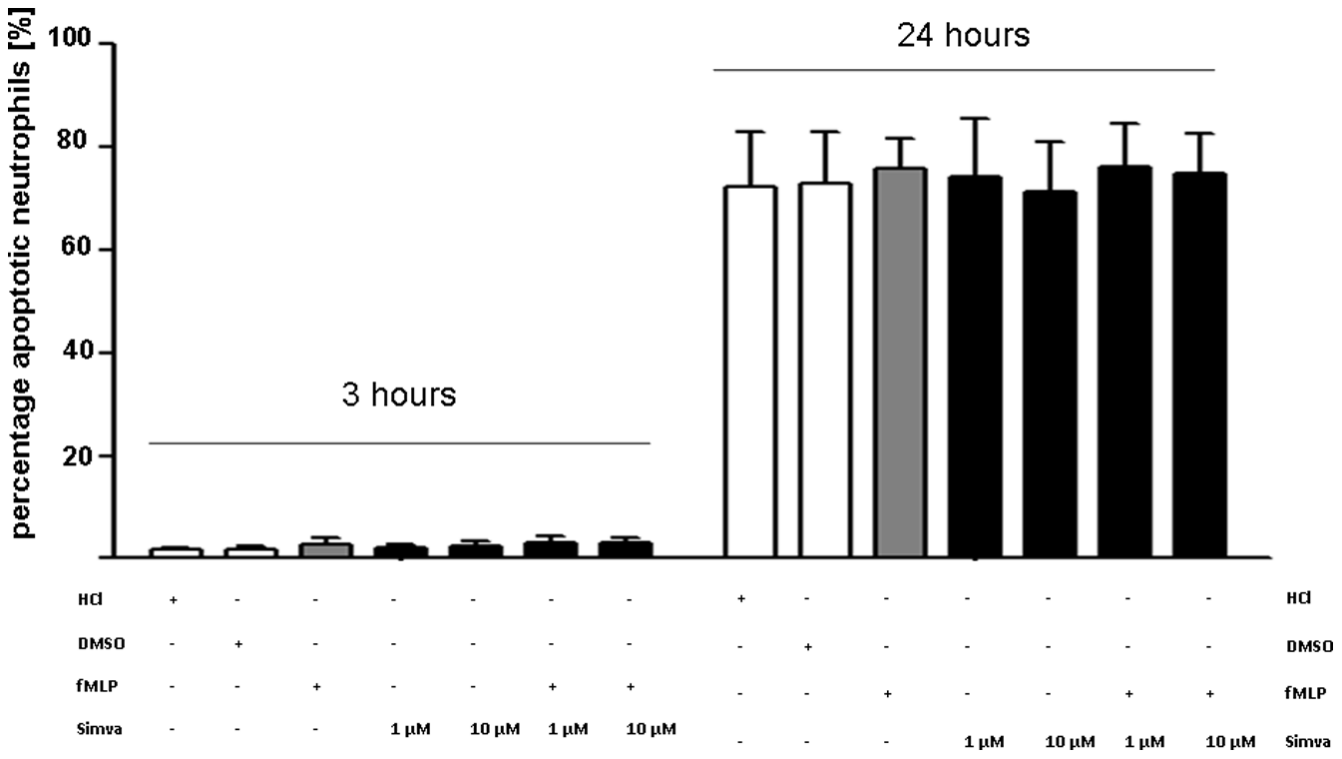
Simvastatin does not affect neutrophil apoptosis. Apoptosis of neutrophis in presence or absence of simvastatin was measured using Annexin V with flow cytometry after 3h and 24 hours respectively (n = 6; repetition = 3). Statistical significance was tested using one way ANOVA with Newman-Keuls Multiple Comparison test. * indicates significant difference to the control group without neutrophils.

In addition, we analyzed in vitro neutrophil apoptosis in presence of simvastatin. In these experiments simvastatin had no effect on neutrophil apoptosis at 3 or 24 hours independently of the concomitant presence of fMLP (Fig. 7).

## Discussion

Despite all innovation in the intensive care, acute lung injury induced by Gram-negative bacteria is still an unsolved problem. Recruitment of neutrophils is a key event in development of ALI [1], [4] linking to plasma leakage and deterioration of oxygenation. The importance of neutrophils in ALI is supported by studies where lung injury is abolished or reversed by depletion of neutrophils [5], [6]. Much of the neutrophil-dependent ALI is mediated by neutrophil recruitment, ROS release, and granule proteins discharge from activated neutrophils. Hence, treatment of ALI should address the recruitment and degranulation of neutrophils. Although, there is already evidence of the beneficial role of statins in sepsis and ALI, there is little known about the effect of statins on neutrophils.

LPS inhalation mimics human Gram-negative ALI, inducing neutrophil recruitment, pulmonary edema and finally impairment of gas exchange [3]. In our study, we confirmed the causative role of neutrophils in LPS-induced ALI, because neutrophil depletion reduced plasma leakage und histological signs of lung injury. Whereas recent studies showed the beneficial effect of simvastatin treatment before ALI [16], [17], our study revealed the same beneficial effect of statins which were administered after inducing ALI. Hence, these results highlighted the clinical relevance of statin therapy which is at present only supported by small clinical trials [28].

Different mechanisms of endothelial barrier protection by statins have been reported. Endothelial junctions are stabilized due to inhibition of RhoA/Rho kinase pathways [29]–[31]. Beside this protection of endothelial barrier, simvastatin might directly reduce the activation and recruitment of the neutrophils which contribute to the tissue damage in acute lung injury [4]. Consequently, we analyzed the effects of simvastatin on neutrophil activity. The importance of neutrophil infiltration in LPS-induced ALI is substantiated in models, where neutrophil adhesion or migration is impaired. In this context it was shown that lack of CXCR2 or blockade of β_2_-integrins protects from ALI [32], [33]. In our hands, simvastatin prevents intravascular neutrophil adhesion and lung infiltration. As this was recapitulated in an *in vitro* assay in absence of other cell types but in presence of substrates typically involved in neutrophil adhesion and migration we conclude that the *in vivo* effects may to a large part relate to direct interference with neutrophil effector functions.

Although the release of ROS displays an important antimicrobicidal mechanism, overproduction of ROS can cause tissue damage in sepsis and ALI [34]. In animal models of ALI, neutrophil-derived ROS caused lung injury as shown by histological examination and permeability measurements [35], [36]. In addition, it was evidenced that ROS can disrupt intercellular tight junctions of the endothelium by phosphorylation of focal adhesion kinase [37]. Hence, deficiency or blockade of NADPH oxidase prevents from ALI [38]–[40]. Here we show that simvastatin effectively prevents neutrophil ROS production which may at least in part explain beneficial effects of simvastatin in ALI shown here. Consistently, studies demonstrated reduced activation of NADPH oxidase by statins in endothelium and macrophages resulting in reduced release [14], [29]. The two most important findings of this study leading to diminished ALI following statin treatment are reduced neutrophil adhesion and recruitment as well as decreased ROS production. It has previously been shown that statins inhibit small GTPases such as Rho and Rac1 which may serve as mechanistic explanation for our findings [41], [42]. On the one hand, Rho activity is important in cytoskeletal re-arrangement and as such involved in leukocyte adhesion and emigration [43]. On the other hand, statins were shown to block Rac1. Rac1 binds to p67^phox^ and leads to activation of the NADPH oxidase system and subsequent generation of ROS [42].

After activation, simvastatin did not significantly ameliorate the degranulation of neutrophils. The expression of CD11b was reduced after pretreatment with simvastatin, but without statistical significance. In accordance to our results, recent studies showed reduced CD11b expression and inhibition of CD11b-dependent monocyte adhesion to endothelium by HMG-CoA reductase inhibitors [44] Similarly, atorvastatin treatment led to a significantly lower expression of CD40L, CD11b and CD54 on monocytes and neutrophils in patients with coronary heart disease [45]. In a M1 protein induced ALI, simvastatin reduced production of CXC chemokines in the lung as well as up-regulation of CD11b on circulating neutrophils [46]. CD11b and FPRL1 are stored in secretory vesicles, a subset of neutrophil granules easily mobilized after neutrophil activation. Following discharge of this compartment, both receptors are incorporated in the cell membrane, while soluble mediators are released. Azurocidin a protein enriched in secretory vesicles is a potent mediator of neutrophil-derived permeability changes and has previously been suggested to be involved in septic lung edema formation [7], [47]. Hence, the trend towards reduced discharge of secretory vesicles in our study may together with effects on ROS formation provide an explanation for reduced permeability changes in simvastatin-treated mice.

Further to emigration, neutrophils are irreplaceable in bacterial clearance, much of which is mediated by phagocytosis and intracellular bacterial killing [48]. In our hands these functions were not negatively affected by presence of simvastatin. Consistently, it has recently been shown that statins promote release of neutrophil extracellular traps [49], a scaffold for catching and killing bacteria. Hence, the lack of adverse antimicrobial side effects confirms the potential capacity of statins in clinical settings of bacterial ALI. However, in contrast to our results, Fessler et. al. showed negative effects of lovastatin on pulmonary antibacterial host defense after intratracheal instillation of *Klebsiella pneumonia* [19]. These divergent results may caused by using different models of bacterial infection. Whereas we induced sepsis and lung infection by cecal ligation and puncture (CLP), Fessler et. al. used a direct intratracheal instillation of *K. pneumonia.* Moreover, the different used inhibitors of HMG-CoA reductase may also display effects with different intensity. Therefore, transfer of these results to clinical setting has to be done cautiously. However, the majority of the clinical studies revealed an increasing evidence that statins have a beneficial effect on the outcome of infection in humans without disturbed host defense [15].

### Conclusion

Simvastatin reduces recruitment and activation of neutrophils in a model of acute lung injury and hereby displays beneficial effects. Moreover, Simvastatin treatment early after LPS inhalation was protective implicating a potential role for statins in the prevention of ALI.
