# Expression of Concern: Simvastatin Reduces Endotoxin-Induced Acute Lung Injury by Decreasing Neutrophil Recruitment and Radical Formation

**DOI:** 10.1371/journal.pone.0240344

**Published:** 2020-10-15

**Authors:** The PLOS ONE Editors

After this article [1] was published, concerns were raised about similarities between this work and work reported previously in *American Journal of Respiratory and Critical Care Medicine* [2] and *European Respiratory Journal* [3].

Specifically,

1) Similarities were noted between the following figures:

- Scanning electron microscopy (SEM) data in Fig 2A (control) of [1], Fig 1C (ctrl) of [2], and Fig 2b in [3]
- SEM data in Fig 2A (LPS) of [1] and Fig 2d in [3]
- Experimental overview diagram in Fig S1 of [1], Fig E1 of [2], and File S1 of [3]
- Flow cytometry data in Fig S2 of [1], Fig E2 of [2], and File S2 of [3]

The authors confirmed that the SEM panels are identical in the three articles. Regarding the supporting information files, the authors noted that the experimental diagram and flow cytometry figures are very similar, though not identical, because the same experimental workflow and strategy to identify neutrophils were used in all three studies.

2) Quantitative data reported in histograms for some groups (e.g. control, LPS, neutrophil-depleted) appear similar in [1–3].

3) The three related articles [1–3] address closely related research questions and were submitted for publication at the respective journals within a short period of time, however the related articles were not declared when the PLOS ONE manuscript was submitted.

In responding to these issues, the authors provided the following clarifications about the relationship between the studies reported in [1–3] and reuse of animals and data across the three studies:

The studies reported in [1–3] all address the role of neutrophils in LPS-induced acute lung injury, but each article addresses a distinct research question and reports different outcomes:

- The study reported in [1] aimed at understanding the impact of Simvastatin, a HMG-CoA reductase inhibitor, on LPS-induced lung injury. The data show that Simvastatin reduces neutrophil infiltration into the lungs as well as the associated lung damage. Mechanistically, however, simvastatin seems to act differently from Pioglitazone, as it reduces ROS production but does not alter neutrophil integrin expression.
- The study reported in [2] dissected the mechanisms of neutrophil-platelet interplay during neutrophil recruitment in LPS-inflamed lungs. Results of this work identified a role for protein heterodimers and use a peptide to disrupt these heterodimers in order to reduce neutrophil lung infiltration.
- The study reported in [3] aimed at understanding the impact of Pioglitazone, a PPAR-γ activator, on the LPS-induced lung injury. Data from this study revealed that Pioglitazone reduces neutrophil infiltration by directly affecting neutrophil integrin expression. The ability of neutrophils to kill bacteria and to generate ROS was not affected.

The experiments reported in [1–3] used the same animals and, for some outcomes, the same data for the control (ctrl), LPS, and neutrophil depletion groups. These experimental groups were not used to achieve novel information but rather to serve as reference groups for the other experimental interventions examined in each study.

Animal experiments displayed in Figs 1 of all [1–3] were performed concurrently, as was in vitro experimentation to test neutrophil functionality. In an attempt to limit the number of control mice included for these studies, data were pooled for the three studies: for each treatment group an initial experiment was performed including 2–3 mice per control group (ctrl, LPS, neutrophil depletion) side by side with the actual intervention (3–4 mice per group). These control groups were then pooled to serve as controls across the three studies. When the treatment groups were acquired in a second, independent run (4–5 mice), additional 2 LPS mice were included to ensure that the LPS response was similar compared to previous observations. That way, mice displayed as ctrl and neutrophil depletion groups are identical, while mice displayed as LPS group vary by 2 mice per group. For protein measurements in the BAL fluid, we used an albumin ELISA in [1] and a Bradford assay in the 2, 3], hence there are discrepancies in this parameter across articles. BAL elastase activity in [3] shows different values as these samples were measured separately from the elastase measurements performed for [1, 2]. For in vitro adhesion assays to ICAM1, ctrl and fMLP groups are identical between the [1] and [2] as MKEY and Simvastatin were tested concurrently in this assay.

The authors apologize for not having clearly delineated in [1] the relationships between these articles and the samples and data that overlapped between [1–3].

The authors stated that the original underlying data for the *PLOS ONE* study are no longer available.

In light of the undeclared data reuse across publications and the unavailability of underlying data to support the article’s [1] results, the *PLOS ONE* Editors issue this Expression of Concern.

The control and LPS SEM panels in Fig 2A and the images reported in Fig S1 and Fig S2 report material that duplicates or is highly similar to content previously published in [2] by the American Thoracic Society and [3] by the European Respiratory Society (ERS). The content in [2] and [3] is not offered under a CC-BY license. At the time of this notice, the *PLOS ONE* article [1] was republished to remove the images in question and replace in-text citations to these images with citations of the prior publications. Please download the PDF again to view the correct version.
